# Creating an Age-Friendly Workplace for Older Workers and Employers

**DOI:** 10.1093/geroni/igaa057.209

**Published:** 2020-12-16

**Authors:** Lisa Hollis-Sawyer

**Affiliations:** Northeastern Illinois University, Chicago, Illinois, United States

## Abstract

Despite the clear aging trends in the U.S. and global population (e.g., World Health Organization, 2015), there has been a lack of “age audit” tools to evaluate the age-friendliness of workplace environments to facilitate older adults’ positive mental/cognitive health, physical health, social/interpersonal health, and general aging adaptation. The present study did a review of the literature and audit tools across several disciplines (psychology, gerontology, kinesiology, anthropometry, audiology, vision science, human resources management, architecture, and social factors engineering) regarding the assessment and design issues underlying “age-friendliness” in the workplace. Further, the present research pilot-tested a new audit tool in two organizations (educational, industrial). The researcher, in coordination with two independent raters, conducted a content analysis of the different peer-reviewed articles and books across several disciplines and available age audit tools/approaches to identify: (1) current practices in age-friendliness assessments (e.g., “user-friendliness” of audit tools for practitioners), (2) potential biases/limitations in age assessments (e.g., “decline/decrement” aging perspective), and (3) “gaps” in evaluations to create a more holistic evaluation approaches. The following conclusions were made: (1) most assessments focused on one factor of functioning (e.g., psychomotor capability), (2) existing tools are limited in options and functionality for daily assessments, (3) most focus on decline and limitations in functioning, and (4) need to design multi-sensory, multi-function assessments reflecting an integrated and coordinated system of sensory, psychomotor, social, and cognitive performance. A holistic model of the outcomes for workplace design “fit” interventions to create more aging-friendly workplaces based upon pilot test results will be presented.

